# scHilda: Hierarchical Integration of LLM with KG database for single cell type annotation

**DOI:** 10.1371/journal.pcbi.1014291

**Published:** 2026-05-11

**Authors:** Yilang Li, Yidi Sun, Aoyun Geng, Junlin Xu, Yajie Meng, Feifei Cui, Leyi Wei, Quan Zou, Xiulai Li, Zilong Zhang

**Affiliations:** 1 School of Cyberspace Security (School of Cryptology), Hainan University, Haikou, China; 2 School of Computer Science and Technology, Hainan University, Haikou, China; 3 School of Computer Science and Technology, Wuhan University of Science and Technology, Wuhan, Hubei, China; 4 School of Computer Science and Artificial Intelligence, Wuhan Textile University, Wuhan, Hubei, China; 5 Centre for Artificial Intelligence driven Drug Discovery, Faculty of Applied Science, Macao Polytechnic University, Macao Special Administrative Region, China; 6 Institute of Fundamental and Frontier Sciences, University of Electronic Science and Technology of China, Chengdu, China; Guangxi University, CHINA

## Abstract

Cell type annotation in single-cell RNA sequencing is a critical bottleneck, with existing automated methods facing limitations in accuracy, interpretability, and generalization to novel cell types. Although Large Language Models (LLMs) have recently shown potential in single-cell annotation, they are prone to inherent “hallucinations”. Furthermore, a critical challenge is utilizing imperfect and potentially noisy external knowledge bases in a principled and robust manner to effectively constrain and enhance the LLM’s reasoning capabilities. To address this, we propose scHilda, a novel framework designed to tackle this challenge. It deeply integrates an external Knowledge Graph into the LLM’s reasoning process and employs a hierarchical arbitration annotation strategy. This strategy ﬁrst identiﬁes major cell lineages with the support of a global knowledge base and then dynamically retrieves focused subgraph domain information related to that lineage to precisely resolve cell subtypes. This dynamic knowledge-enhanced reasoning mechanism effectively constrains the LLM’s decision space, reduces the risk of hallucination, and mitigates potential misguidance from knowledge base deﬁciencies. Tests on multiple benchmark datasets show that scHilda outperforms existing methods, achieving state-of-the-art (SOTA) performance. Notably, scHilda demonstrates exceptional robustness when handling complex mixed samples and enables lower-cost lightweight LLMs to achieve annotation performance close to that of top-tier models. Furthermore, rigorous statistical evaluations, alongside detailed interpretability case studies and query complexity analyses, validate the framework’s efficiency and transparent decision-making. By deeply integrating the reasoning power of LLMs with structured biological knowledge, scHilda not only improves the accuracy and interpretability of cell annotation but also provides a new paradigm for building the next generation of trustworthy biological AI systems.

## Author summary

Identifying the exact types of cells within a sample is fundamental to advancing biological and medical research. Recently, large language models (LLMs) have shown great promise in automating this complex annotation process. However, these models can sometimes hallucinate, confidently generating incorrect biological answers. While previous methods attempted to fix this by using external databases to double-check the LLM’s output, but which rigid or imperfect databases will sometimes restrict the model’s potential and misguide its decisions. In this study, we introduce scHilda, a novel computational framework that solves this dilemma. Instead of using databases merely for post-hoc validation, scHilda deeply integrates a structured biological knowledge graph directly into the LLM’s step-by-step reasoning process. It guides the LLM to first reliably identify broad cell lineages and the dynamically zooming in on specific cellular subtypes using focused local knowledge. Our comprehensive evaluations demonstrate that scHilda significantly outperforms existing tools in accuracy. It remains exceptionally robust when analyzing complex, mixed biological samples and enables researchers to achieve top-tier annotation results using more affordable, lightweight LLMs. Ultimately, scHilda provides a highly transparent, efficient, and trustworthy LLM-based assistant for scientists exploring cellular diversity.

## Introduction

As a key downstream analysis task in single-cell RNA sequencing (scRNA-seq), the evolution of methods for cell annotation has always aimed to strike a better balance between automation, accuracy, and the integration of biological knowledge. Early manual annotation, which relied on expert experience, was time-consuming, laborious, and highly subjective [1].

To overcome these bottlenecks, automated methods based on deep learning have been widely explored. Supervised deep learning models have evolved from Multi-Layer Perceptrons (MLP), such as ACTINN [2], to Convolutional Neural Networks (CNN) like scDeepInsight [3], and recently to Transformer-based architectures like scBERT [4]. While these models significantly improved annotation efficiency, their performance heavily depends on large-scale, high-quality labeled reference data. To alleviate the issue of data scarcity, semi-supervised learning paradigms were introduced. Methods utilizing deep generative models (e.g., scANVI [5]), domain adversarial networks (e.g., scNym [6]), pseudo-labeling (e.g., scSemiPLC [7]), and graph regularization (e.g., CALLR [8]) attempt to leverage vast amounts of unlabeled data. However, their robustness often degrades sharply when confronted with real-world biological noise and imperfect reference labels. Furthermore, to explicitly capture high-order topological relationships such as gene regulatory networks and cell-cell interactions, Graph Neural Networks (GNNs) [9] like scDeepSort [10], scMGCN [11], and scMCGraph [12] have been developed to model cellular microenvironments.

Despite these architectural advancements, traditional deep learning frameworks share some fundamental limitations. For instance, these methods are highly sensitive to training data distributions and lack zero-shot reasoning capabilities, often misclassifying rare or novel cell types in the long tail. Additionally, most models fail to provide semantic, biologically meaningful explanations for their predictions, severely hindering interpretability.

In recent years, the rise of Large Language Models (LLMs) has provided a new paradigm for cell annotation [13,14]. The work of Hou and Ji [15] ﬁrst demonstrated the zero-shot/few-shot annotation potential of GPT-4 in this ﬁeld, with their resulting tool, GPTCelltype, showing high consistency with expert annotations. Although LLMs can generate natural language explanations—offering a different form of interpretability compared to the entirely black-box nature of traditional deep learning—their internal decision-making mechanics remain fundamentally opaque. Without structured constraints, LLMs are prone to “hallucinations” [16]; they may generate confident but biologically incorrect reasoning and labels, which can be severely misleading for research. Relying solely on an LLM’s ungrounded text generation is insufficient for rigorous biological interpretation. To address this, scHilda improves interpretability by grounding the LLM’s natural language reasoning in explicit, traceable evidence retrieved from a structured Knowledge Graph.

To mitigate the hallucination problem in LLMs, CellTypeAgent [17] attempted to use external biological databases like CellxGene [18] to score and ﬁlter the LLM’s output, using empirical data for fact-checking the LLM’s abstract reasoning. However, this post-hoc validation strategy introduces a deeper “Impedance Mismatch” problem: the conﬂict between a powerful, ﬂexible reasoning engine and an external knowledge source that may be incomplete or contain errors. For example, if an LLM correctly infers a novel cell subtype based on its vast pre-training data, but this subtype is undeﬁned or incorrectly labeled in the validation database, the validation step will erroneously discard the correct result and instead choose a common but incorrect type from the database. Therefore, relying on external databases for mandatory validation is a high-risk strategy that no only stiﬂes new biological discoveries but also caps the model’s performance at the quality level of the validation database. Furthermore, having the LLM perform self-scoring has also been shown to be unreliable, as the model often exhibits overconﬁdence in its own answers, failing to provide effective differentiation.

To overcome the limitations of the above methods, we propose scHilda, a novel framework aimed at solving the “impedance mismatch” problem by re-architecting the interaction between external knowledge and LLMs. The core philosophy of scHilda is that external knowledge should not act as a “Post-hoc Veriﬁer” for retrospective review, but as a “Co-processor” deeply integrated into the reasoning process, with the ﬁnal decision made by the LLM with the support of sufﬁcient contextual information. Speciﬁcally, scHilda incorporates two core designs:

1) Hierarchical Annotation with Arbitration: This design is inspired by the ﬁndings of scHDeepInsight [3], which demonstrated that following the biological hierarchy of cells can improve annotation accuracy. scHilda first leverages the LLM’s intrinsic knowledge to propose candidate major cell lineages. It then definitively determines the true major cell type with the aid of evidence retrieved from a global knowledge base. Finally, it dynamically retrieves the focused subgraph domain related to that specific lineage to identify the precise cell subtype. This structured process effectively constrains the LLM’s reasoning path and reduces the risk of hallucination.
2) KG-Enhanced Reasoning: scHilda constructs a comprehensive biological Knowledge Graph (KG) [19,20] from public datasets like the Cell Ontology [21] and gene pathway databases [22–24], containing millions of nodes (e.g., 3,129 cell types, 23,280 genes) and hundreds of thousands of relationships (e.g., IS_A, PARTICIPATES_IN). For major types, scHilda performs a comprehensive global query of the database based on marker genes. When the annotation focuses on subtypes, scHilda locks onto the subgraph domain of the identiﬁed major type through hierarchical relationships, ignoring thousands of irrelevant relationship noises. This dynamic retrieval mechanism signiﬁcantly improves search efﬁciency, which is also a practical application of the philosophy behind advanced Retrieval-Augmented Generation (RAG) [25] frameworks like LightRAG [26]. In this context, our external knowledge transforms from a rigid validation tool into a ﬂexible reasoning aid, fundamentally resolving the “impedance mismatch” problem.

This novel architecture enables scHilda to outperform existing methods on multiple benchmark datasets, achieving State-of-the-Art (SOTA) annotation performance. Its advantages are particularly evident in its exceptional robustness and accuracy when dealing with complex cell types and challenging scenarios like incomplete external databases. And we also require the LLM to provide evidence for its reasoning to enhance interpretability. Under this framework, some less parameter-intensive, lightweight LLMs can also achieve performance comparable to advanced LLMs. By deeply synergizing the powerful few-shot reasoning capabilities of LLMs with a high-quality external knowledge graph, scHilda not only fundamentally solves the “impedance mismatch” problem but also provides a brand-new design paradigm for building the next generation of trustworthy and interpretable biomedical AI systems.

## Methods

### The framework of scHilda

**Fig 1:**
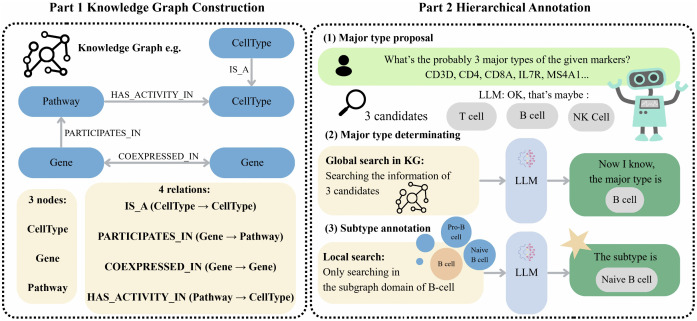
The scHilda framework, divided into two parts. Part 1 (Knowledge Graph Construction): Defines the KG structure, containing three node types and four directed relationships. Part 2 (Hierarchical Annotation): Illustrates the core three-stage workflow. Major type proposal: The LLM proposes 3 probable major type candidates (e.g., T cell, B cell, NK Cell) based on input markers (e.g., CD3D, CD4, CD8A, etc.). Major type determining: The system performs a global KG query on the 3 candidates. The LLM integrates the retrieved evidence to determine the single major type (e.g., B cell). Subtype annotation: The system performs a local search within the confirmed major type’s (e.g., B cell) subgraph domain. The LLM then uses this focused information to annotate the final subtype (e.g., Naive B cell).

As illustrated in Fig 1, scHilda is a framework integrating Knowledge Graphs with Retrieval-Augmented Generation to enhance the accuracy of cell annotation across different tissues through hierarchical LLM-based annotation. In the system initialization stage, we first constructed a biological KG based on prior biological knowledge, leveraging structured information to help the large language model mitigate “hallucinations” issues and improve reasoning reliability. During the annotation process, the LLM first proposes candidate major cell types based on its intrinsic knowledge. It then determines the final major type in conjunction with information retrieved from the global knowledge base. Subsequently, scHilda retrieves the subgraph domain corresponding to the confirmed major type by following hierarchical relationships within the knowledge base. Finally, the LLM integrates contextual information from the subgraph domain to infer the cell subtype, producing the final annotation result.

### Construction of the scHilda Biological Knowledge Graph

The scHilda knowledge graph (as shown in Fig 1 Part1) is built around three main types of nodes: Gene, Pathway, and CellType. These nodes are interconnected through four types of directed relationships: IS_A, PARTICIPATES_IN, COEX- PRESSED_IN, and HAS_ACTIVITY_IN. The entire knowledge graph contains 23,280 Gene nodes, 3,795 Pathway nodes, and 3,129 CellType nodes, with over 370,000 relationships.

#### IS_A (CellType → CellType).

This relationship is derived from the Cell Ontology (CL) [21] and deﬁnes the hierarchical classiﬁcation structure among cell types. This relationship enables the model to perform hierarchical reasoning from major types (e.g., “lymphocyte”) to speciﬁc subtypes (e.g., “cytotoxic T lymphocyte”).

#### PARTICIPATES_IN (Gene → Pathway).

Data for this relationship is integrated from the Gene Ontology (GO) [22] Consortium and the Reactome [23] pathway database [24]. It connects genes to the biological pathways they functionally participate in. This relationship provides a functional context for genes, allowing the LLM to recognize sets of marker genes as coordinately activated biological pathways. Subsequently, for each dataset introduced into the KG, we need to reconstruct the COEXPRESSED_IN and HAS_ACTIVITY_IN relationships.

#### COEXPRESSED_IN (Gene → Gene).

This relationship captures signiﬁcant co-expression patterns of gene pairs within a speciﬁc cell type. We quantify the co-expression strength between any two genes, X and Y, by calculating the Pearson product-moment correlation coefﬁcient (r):


$$\begin{array}{c} \begin{array}{c} r=\frac{\sum_{i=\text{1}}^{n}(x_{i}-\bar{x})(y_{i}-\bar{y})}{\sqrt{\sum_{i=\text{1}}^{n}(x_{i}-\bar{x}{)}^{\text{2}}}\sqrt{\sum_{i=\text{1}}^{n}(y_{i}-\bar{y}{)}^{\text{2}}}} \end{array} \end{array}$$ (1)

where: n is the total number of cells of a speciﬁc cell type. $x_{i}$ and $y_{i}$ are the log-normalized expression values of gene and gene in cell i, respectively. $\bar{x}$ and $\bar{y}$ are the average expression values of gene X and gene Y across all n cells, respectively. When the correlation coefﬁcient between two genes exceeds a statistically signiﬁcant threshold (e.g., |r| > 0.8, False Discovery Rate (FDR) < 0.05) [27], a COEXPRESSED_IN edge is created between them. This combined threshold is intended to build a high-ﬁdelity co-expression network, ensuring that the included relationships have both strong biological synergy (|r| > 0.8) and strict statistical signiﬁcance (FDR < 0.05) [27].

#### HAS_ACTIVITY_IN (Pathway → CellType).

This relationship represents the activity of gene pathways. To quantify the activity of a given pathway (i.e., a gene set) in each single cell, we employ the AUCell algorithm [28]. The AUCell algorithm quantiﬁes the activity of a speciﬁc gene set in a single cell by calculating the Area Under the “recovery Curve” (AUC). First, all genes within a single cell are ranked by their expression levels from high to low. Then, for a given gene set, a “recovery curve” is plotted by moving down this ranked list, where the Y-axis represents the number of genes from the gene set that have been hit up to the current rank. Finally, the area under this curve (AUC) within a preset ranking threshold (usually the top-expressing genes) is calculated. This AUC value serves as the activity score for that gene set in that cell, with higher scores indicating stronger activity. The AUCell score is the integral of a gene set’s Recovery Curve over the gene expression ranking list.


$$\begin{array}{c} \begin{array}{c} AUCell\left(S,R_{c}\right)=\int_{\text{0}}^{T}f\left(k,S,R_{c}\right)\;dk\; \end{array} \end{array}$$ (2)

where the recovery function f (k, S, Rc) is deﬁned as:


$$\begin{array}{c} \begin{array}{c} f\left(k,S,R_{c}\right)=\frac{\left|\left\{g\in S|rank\left(g,R_{c}\right)\leq k\right\}\right|}{\left|S\right|}\; \end{array} \end{array}$$ (3)

**S (Gene Set).** A list of genes for a biological pathway. c (Cell): A single cell. Rc (Gene Ranking): The ranking of all genes in cell by expression from high to low. k (Rank): The speciﬁc position of a gene in the ranking list Rc. f (k, S, Rc): The recovery function, representing how many genes from set have appeared up to rank k. T (Threshold): The ranking threshold, which is the upper limit for calculating the AUC area, meaning only genes ranked up to T are considered. AUCell typically defaults to the top 5% of total genes as the threshold, aiming to focus on the most actively expressed genes. If the median AUCell score of a pathway across all cells of a certain cell type is signiﬁcantly higher than in other cell types, a HAS_ACTIVITY_IN edge is established between that Pathway and CellType. In practice, we use the Wilcoxon rank-sum test for quantitative judgment. When the pathway score distribution in the target cell type is statistically signiﬁcantly higher than in the remaining cells (p < 0.05 after multiple testing correction), this relationship is established.

### Hierarchical reasoning and annotation strategy of scHilda

The core workﬂow of scHilda (as shown in Fig 1 Part2) follows a phased, coarse-to-ﬁne annotation strategy that deeply integrates the reasoning capabilities of the LLM with structured evidence from the knowledge graph. The process mainly includes three key stages:

#### Stage 1.

LLM-led Proposal of Major Cell Types Leveraging the LLM’s internalized biological knowledge, a candidate list of the most 3 probable major cell types is rapidly generated for each cell cluster. The list of marker genes for each cell cluster is used as input. The script constructs a structured prompt for each batch of cell clusters and submits it to the LLM.

You are an expert in single-cell transcriptomics. Your task is to act as a primary annotator.

For each provided cell cluster index and its marker genes, you must propose the 3 most probable broad cell lineages (major types). Your reasoning should be based on your deep biological knowledge. The candidates should be ordered from most likely to least likely.

This step generates a list of 3 candidate major cell types for each cell cluster, with each candidate including a name, ontology ID, and reasoning.

#### Stage 2: Knowledge Graph Integration to Determine the Major Type.

For each candidate generated in Stage 1, the system performs a global query of the knowledge graph to collect qualitative evidence (ontology structure, functional pathway associations, co-expression network support). Subsequently, all candidates, their reasoning, and the corresponding KG evidence are integrated into a second prompt.

You are a senior computational biologist acting as the final arbiter for a challenging cell type annotation case. An initial analysis has yielded several hypotheses. Your task is to critically evaluate all available qualitative evidence from the Knowledge Graph to make a definitive judgment on the most likely major cell type.

The Knowledge Graph contains curated, symbolic knowledge about cell types. Your decision should consider the provided KG summaries for each candidate.

The ﬁnal output is a unique and conﬁrmed major cell type name and ID.

#### Stage 3: Knowledge Graph Subgraph Domain Retrieval and Final Annotation.

Once the major type is conﬁrmed, the system performs a focused subgraph query in the knowledge graph. This query is restricted to the subgraph composed of the conﬁrmed major type and its descendants, retrieving all cell subtypes that have functional pathway associations with the input marker genes. This ﬁlters out thousands of relationships and nodes irrelevant to that cell lineage. The subgraph search does not query the COEXPRESSED_IN relationship to avoid interference from this less speciﬁc relationship in subtype determination.

You are a cell annotation specialist. The major cell type has been confidently identified.Your task is to determine the most specific subtype based on the provided focused evidence from a knowledge graph.

The ﬁnal output includes the ﬁnal cell type name, ID, reasoning, and relevant evidence, representing the ﬁnal annotation conclusion for that cell cluster. The output reasoning, combined with the knowledge graph evidence, provides a complete explanatory path for the annotation result, enhancing the model’s interpretability and avoiding the “black-box” problem. This dynamic retrieval mechanism is designed to resolve the “impedance mismatch” between the LLM’s internal knowledge and external structured knowledge. By positioning the knowledge graph as a “consultant” to guide reasoning rather than the ﬁnal “arbiter”, this framework utilizes external knowledge while preserving the LLM’s comprehensive judgment capabilities.

### Performance evaluation

We used the “Agreement Score” metric proposed by Hou and Ji [15] to evaluate the accuracy of cell type annotation. This metric is speciﬁcally designed to account for the hierarchical structure of the Cell Ontology, categorizing each prediction’s comparison with the ground truth label into three types:

#### Fully Match (Score = 1.0).

The predicted cell type is identical to the ground truth label or is an exact synonym in the Cell Ontology.

#### Partially Match (Score = 0.5).

The predicted cell type and the ground truth label share a direct parent node or are sibling nodes in the Cell Ontology (e.g., ground truth is “CD4+ T cell,” prediction is “CD8+ T cell”).

#### Mismatch (Score = 0.0).

The predicted cell type and the ground truth label belong to different major lineages (e.g., ground truth is “T cell,” prediction is “ﬁbroblast”).

The ﬁnal agreement score for a dataset is the average of the scores for all annotated cell clusters. Compared to traditional ﬂat metrics like accuracy, this metric provides a more biologically meaningful measure of annotation effectiveness because it penalizes different types of errors differently.

### Analysis of knowledge graph query complexity

To comprehensively evaluate the computational efficiency of the scHilda framework, we analyzed the knowledge graph query complexity at different annotation stages. Considering that our complete knowledge graph contains over 30,000 nodes (23,280 genes, 3,795 pathways, and 3,129 cell types) and over 370,000 relationship edges, a naive full-graph query approach is not only computationally expensive but also highly susceptible to introducing massive noise. Furthermore, querying such a vast network via an online Neo4j backend incurs significant network I/O latency.

scHilda circumvents this issue by significantly reducing the search space through a hierarchical retrieval strategy. In Stage 2 (Determining Major Type), the query complexity is reduced to O(1) index lookups strictly targeting the 3 candidate major types proposed by the LLM. In Stage 3 (Subtype Annotation), the complexity decreases exponentially compared to a full-graph search. By restricting the query to the specific subgraph domain of the confirmed major type and explicitly excluding the highly dense COEXPRESSED_IN edges, the subgraph retrieval directly filters out thousands of irrelevant cell lineages and relationships. Empirical measurements on our benchmark datasets show that, on average, the subgraph query in Stage 3 involves only approximately 150 nodes and 230 edges, which is a massive reduction from the full graph’s 370,000 + edges.

This multi-stage mechanism for reducing query complexity ensures that the context provided to the LLM is highly precise with extremely low token consumption. It effectively prevents the model from being overwhelmed by irrelevant graph noise, thereby further mitigating “hallucinations” while enabling the entire batch annotation process to be completed efficiently within 3–5 minutes.

### Evaluation datasets

The benchmark datasets used in this study were collected from two public sources:

**From Published Literature.** We extracted expert-manual annotated cell types and their corresponding marker gene lists, which serve as the “gold standard,” from a series of original research articles [29–34].

**From Large-scale Research Projects.** We also obtained authoritative cell annotation information and marker gene sets from ofﬁcial data portals such as the Azimuth project of the Human BioMolecular Atlas Program (HuBMAP) [35,36].

In the public datasets used in this study, most datasets provided approximately ten biomarker genes after preprocessing. To ensure fairness and consistency across different methods and datasets, we uniformly adopted ten marker genes as the input parameter for all methods in the benchmark performance comparison. At the same time, we also evaluated the impact of the number of marker genes on the performance of large language models. Furthermore, to validate the robustness and effectiveness of our approach, we incorporated additional datasets that contained a larger number of marker genes and investigated the performance of scHilda under these expanded input conditions (see Section 3.8 for details).

### Conﬁguration and setup

Knowledge Graph Backend: The knowledge graph is stored in a Neo4j graph database and interacted with via the ofﬁcial Python neo4j driver package.

**Parallel Computing and Time.** The entire process is efﬁciently parallelized using Python’s concurrent futures module. We default to 64 parallel workers, with each batch containing 4 cell clusters. All datasets can be annotated within 1–3 minutes.

**API and Cost.** We conducted tests on both DeepSeek-V3.2 [37] and OpenAI o3 [38] model. Under our framework, DeepSeek-V3.2 can achieve results close to or even surpassing o3 on some datasets. Therefore, we default to DeepSeek-V3.2, which can signiﬁcantly reduce model expenses, with the average cost per cell annotation being $0.001.

**Interpretability.** This model provides interpretability. If this output is turned off, the cost can be further reduced. Tests have shown that performance is not affected at all.

**Inference Parameters.** To ensure a fair comparison and strictly control for variables, all key inference parameters were aligned across the evaluated methods (scHilda, CellTypeAgent [13], and GPTCellType [11]). Specifically, unless otherwise specified in the ablation studies, the number of input marker genes was uniformly fixed at 10 for all methods, and the number of proposed candidate cell types (Top-k) for both scHilda and CellTypeAgent [13] was consistently set to 3. Furthermore, during our experimental phase, the API temperature for the gpt5-nano model [35] was locked at 0.5 by the vendor. To maintain strict experimental consistency, the temperature parameter was uniformly set to 0.5 for all models across all methods.

## Results

### scHilda Demonstrates SOTA on multiple benchmark datasets

**Fig 2:**
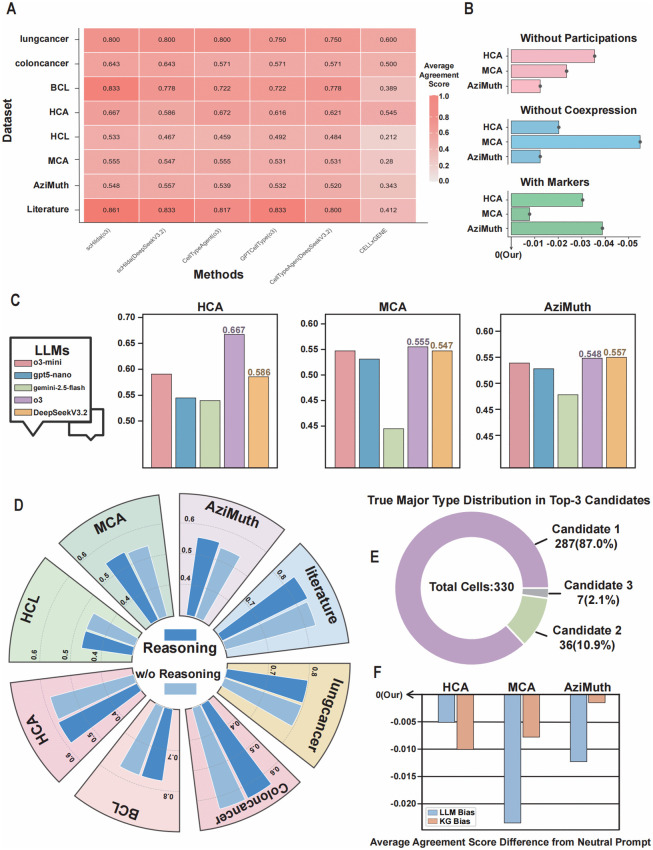
scHilda Performance Evaluation and Ablation Studies. (A) Comparison of annotation agreement scores between scHilda and various existing methods across eight benchmark datasets, showcasing its SOTA performance. (B) Ablation study of different relationship searching types in the knowledge graph, verifying the signiﬁcant contributions of pathway (PARTICIPATES_IN) and co-expression (COEXPRESSED_IN) relationships to annotation accuracy and the negative impact of the Marker relationship on the model. (C) Performance on LLMs of different scales, indicating that the scHilda framework effectively ensures a performance baseline, allowing lightweight models to achieve results close to top-tier models. (D) Comparison of model performance with and without explainability output (reasoning and evidence), showing that disabling this feature can reduce costs with almost no impact on accuracy. (E) The major type distribution in Top-3 candidates, showing the necessity of the major type determination. (F) The impact of different prompt strategies (LLM-biased, KG-biased, neutral) on performance, demonstrating that both LLM-biased and KG-biased approaches show a signiﬁcant drop in performance compared to the neutral one.

To comprehensively evaluate the performance of the scHilda framework, we conducted rigorous comparative experiments on eight widely used public single-cell transcriptomic datasets, using both the o3 [38] and the Deepseek-V3.2 [37] large language models as backbones. The competing methods included our reproductions of CellTypeAgent [17] and GPTCellType [15] based on the o3 model, as well as a baseline approach that performs annotation solely through the CellxGene [18] database. As shown in Fig 2A, regardless of whether o3 or the more lightweight Deepseek-V3.2 model was used, scHilda achieved higher Agreement Scores than all other methods across nearly all benchmark datasets, reaching SOTA performance. These results demonstrate that scHilda, through its unique strategy of hierarchically integrating a knowledge graph with a large language model, attains both high accuracy and strong generalization across data derived from different tissues and species. Notably, we were pleasantly surprised to find that even when employing the more efficient and lightweight Deepseek-V3.2, scHilda maintained excellent annotation performance—highlighting the framework’s flexibility and scalability across different model architectures.

Furthermore, to rigorously validate the robustness of scHilda’s performance improvements across diverse biological contexts, we conducted statistical significance tests and calculated effect sizes (Cohen’s d) based on the average Agreement Scores in model of o3. By treating the 8 independent benchmark datasets—which encompass different tissues, sequencing platforms, and species—as independent macro-observation samples, we performed a cross-dataset statistical analysis (Table 1).

**Table 1:** Statistical Significance and Effect Sizes of scHilda vs. Baseline Methods.

Methods	Mean Diff	Cohen’s d	p-value
vs GPTCellType	+0.049	1.60	0.0027
vs CellTypeAgent	+0.038	0.87	0.0431

### Case study for the interpretability of scHilda

To concretely illustrate how scHilda achieves transparent interpretability, we present a complete annotation case for Cluster 105 from the Azimuth dataset. The input marker genes were *PLXDC2, SET—SPP1, DOCK4, SFMBT2, FRMD4A, SLC9A9, ITPR2, DOCK8, APBB1IP,* and *IPCEF1*. The ground truth label for this cluster is “microglial cell”.

During the major-type determination stage, the LLM evaluates the evidence for three candidates:

Step 1: Assess Evidence Quality. I compared the KG summaries for all three candidates. Endothelial cell and Macrophage each have 1 pathway linking the supplied markers, while Microglial cell has none. All have equal ontology standing, and none shows co-expression links, so pathway support is the only quantitative differentiator supplied by the KG.

Step 2: Examine Marker Specificity. Among the provided genes, PLXDC2, DOCK4 and ITPR2 are indeed seen in angiogenic/tip endothelial cells, but the remaining SET—SPP1, DOCK8, APBB1IP, IPCEF1 are classic myeloid/leukocyte genes. DOCK8 and APBB1IP are especially immune-restricted and rarely found in endothelial cells, tipping the balance toward a myeloid lineage. Microglia are a specialized Central Nervous System (CNS)-resident macrophage subset and normally co-express TMEM119, P2RY12 or CX3CR1, none of which appear in the marker list, making a generic macrophage identity more plausible than microglia.

Step 3: Reconcile KG Pathway Support. Both Endothelial cell and Macrophage have one curated pathway hit, but the endothelial-linked genes are fewer (3/7) and not fully specific, whereas four immune-biased genes strongly favor macrophage biology. Microglial cell lacks any KG-documented pathway support for these markers.

Step 4: Final Verdict. Weighing the higher proportion and specificity of myeloid markers together with equal or better KG pathway backing, the cluster is best classified at the major-type level as Macrophage (CL:0000235).

In the subsequent subtype annotation stage, the system retrieves the focused local KG subgraph for the Macrophage lineage:

Building upon the confident identification of the major cell type (Macrophage), I further evaluated the focused evidence provided by the knowledge graph (KG) for the child nodes of this lineage. The targeted KG search revealed a distinct curated pathway link connecting the marker genes SET—SPP1 and APBB1IP directly to ‘microglial cell’. The KG connectivity for this subtype successfully anchors the broad macrophage identity down to this CNS-resident subtype. In the absence of conflicting evidence supporting other macrophage subtypes (e.g., other tissue-resident macrophages), the most specific and evidence-backed annotation, driven by this crucial KG pathway support, is microglial cell (CL:0000129).

“positive_markers”: [“SET—SPP1”, “APBB1IP”]

This case clearly demonstrates how scHilda’s reasoning narrative is not a black box, but a traceable explanation path that dynamically integrates intrinsic biological logic with external structured KG evidence to resolve conflicting signals.

### scHilda maintains excellent performance on lightweight LLMs

We further investigated the performance of scHilda when using LLMs of different scales and capabilities as its reasoning core. The experimental results, as shown in Fig 2C, indicate that when using larger, more powerful models (like o3), scHilda’s performance correspondingly reaches its peak. This suggests that the design of scHilda can effectively leverage advancements in cutting-edge LLMs. Notably, we speciﬁcally evaluated a series of smaller, lower-API-cost lightweight models, such as gemini-2.5-ﬂash [39], gpt5-nano [40], and o3-mini [41]. The results show that although the ﬁnal performance of these models is slightly lower than o3, the difference is very small. This ﬁnding reveals a key advantage of the scHilda framework: by providing a reasoning scaffold for the LLM through a structured knowledge graph, it effectively guarantees a performance baseline for annotation. Even when using lower-cost lightweight models, scHilda can achieve high-quality annotation results close to those of top-tier models, which offers a highly attractive application prospect for large-scale or budget-sensitive research.

Additionally, our evaluations reveal a positive correlation between the inherent reasoning capability of the LLMs and final annotation accuracy. To deeply understand the performance gap between the SOTA model (o3) and the highly cost-effective model (DeepSeek-V3.2), we conducted a detailed error decomposition. Analysis indicates that for both models, the vast majority of errors occur during the major lineage decision stage. While DeepSeek-V3.2 demonstrates reasoning comparable to o3 in most scenarios, its errors primarily manifest in three distinct patterns, and we choose three clusters in the dataset Azimuth as examples:

**Over-extrapolation.** In Cluster 4 of the Azimuth-Adipose dataset (True label: Monocyte), both models correctly assessed the KG evidence. However, o3 correctly adhered to the provided candidate scope, concluding: *“Integrating the neutral KG evidence with the high specificity of the observed marker set, the most plausible major cell type is monocyte (CL:0000576).”* In contrast, DeepSeek-V3.2 recognized *VCAN* and *FCN1* as highly specific to the classical monocyte subset. Lacking KG subgraph support for the subtype level at this stage, it bypassed the prompt’s hierarchical constraints and self-generated a finer-grained child node, stating: *“Integrating the KG’s validation of the monocyte lineage with the exquisite specificity of the marker profile, the most accurate and definitive cell type assignment is Classical Monocyte (CL:0002057).”* This over-extrapolation led to a mismatch with the major label.

**Hallucination.** In Cluster 24 of the Azimuth-Bone Marrow dataset (True label: Plasma cell), both models executed flawless biological reasoning, correctly identifying *SDC1* and *TNFRSF17* as definitive curated markers for terminally differentiated plasma cells. However, in the final output, while DeepSeek-V3.2 output the correct literal name, it hallucinated an incorrect Ontology ID: *“Based on the conclusive marker-to-cell-type linkages supported by the Knowledge Graph, the major cell type is definitively identified as Plasma Cell (CL:0000980).”* The correct ID assigned by o3 was CL:0000786.

**Premature Conclusion.** In Cluster 23 of the Azimuth-Bone Marrow dataset (True label: Granulocyte monocyte progenitor), the marker list mixed early primary-granule enzymes with a lack of mature markers. o3 successfully resolved this conflict, noting that *“the absence of terminal markers... point most specifically to an early granulocyte progenitor.”* Conversely, DeepSeek-V3.2 exhibited an attention bias toward dominant hallmark genes (*PRTN3* and *ELANE*). It skipped the conflict resolution step regarding missing terminal markers and prematurely concluded: *“The robust primary-granule signature completely supersedes the less specific alternative hypotheses... Relying on the definitive hallmark genes present in the profile, the most likely major cell type is Neutrophil (CL:0000775).”*

The full, step-by-step reasoning traces illustrating these three comparative examples are provided in **Section 1 in** S1 Text**, Differences in reasoning traces between o3 and DeepSeek V3.2**.

### Guiding LLM-KG interaction through prompt engineering

The work of CellTypeAgent [13] suggested that introducing an external knowledge base could lead to “conﬁrmation bias” in LLMs, causing them to over-rely on the knowledge base information while ignoring their internalized pre-trained knowledge, thus affecting judgment accuracy. We demonstrated through a series of prompt ablation experiments that this potential bias can be effectively circumvented with careful Prompt Engineering. In scHilda’s standard prompt, we use neutral and critically-minded wording, asking the LLM to treat the evidence provided by the knowledge graph as a “reference” rather than “absolute truth.” As shown in Fig 2F, this setup achieved the best results. We then designed two control experiments:

#### Forced reliance on the knowledge base.

We changed the prompt to strictly require the LLM to ‘be based on the evidence from the knowledge graph.’ The results showed a significant decrease in annotation accuracy. This occurs because the LLM can be misled by the inherent sparsity or incompleteness of the external database. For instance, in Cluster 19 of the Azimuth-Bone Marrow dataset (True label: Progenitor B cell), the LLM’s internal knowledge correctly recognized markers like *VPREB1* and *DNTT* as indicating an early B cell developmental stage. However, because the KG only contained curated pathways for the generic B cell and lacked specific links for pre-B cell, the model was forced to suppress its accurate internal knowledge and retreat to a broader classification. It concluded: *“Because the task is to decide on the most likely major cell type, and the KG provides its only positive functional pathway support to ‘B cell’, the balance of curated evidence favors the broader category... The absence of pathway/network links for the more specific terms weakens those hypotheses in this context.”* The neutral prompt avoids this failure by allowing the LLM to trust its precise internal identification despite the KG’s sparsity.

#### Forced reliance on the LLM’s own judgment.

We added the instruction ‘...trust your expert judgment to resolve any discrepancies or conflicts.’ In this scenario, the LLM over-relies on its internalized knowledge and dismisses valid KG evidence. For example, in Cluster 30 of the Azimuth-Bone Marrow dataset (True label: Proliferating Natural Killer), the KG successfully retrieved a relevant functional pathway for ‘natural killer cell’ and zero for the incorrect alternatives. However, the LLM developed an unwarranted fixation on the *TRDC* gene, erroneously dismissing the KG’s objective evidence: *“Thus, even though the KG provides a solitary generic pathway for NK cells, the lineage-specific marker TRDC outweighs that weak, non-specific support... the most plausible major cell type for this cluster is ‘gamma-delta T cell’.”* Under the neutral prompt, the model correctly balances the evidence to identify the NK cell.

The full reasoning traces illustrating these prompt engineering failure cases are provided in **Section 2 in S1 Text, Reasoning Traces of Prompt Engineering Failure Cases**.

In summary, the optimal strategy is to position the knowledge graph as a “co-processor” that provides objective but potentially ﬂawed evidence, while guiding the LLM to use its powerful reasoning abilities for critical evaluation and preventing overconﬁdence, thereby achieving the best annotation results.

### Different relationship searing types in the scHilda KG

The scHilda Knowledge Graph (KG) incorporates several innovative biological relationships. To validate the necessity of these relationships, we conducted a series of ablation studies by removing each type of relationship and evaluating its impact on the ﬁnal annotation performance (Fig 2B). The results show that removing either PARTICIPATES_IN (pathway relationship) or COEXPRESSED_IN (co-expression relationship) led to a certain degree of decline in scHilda’s accuracy. This conﬁrms the importance of these two relationships in providing context about cellular functions and gene synergy. It is important to note that the IS_A (ontology hierarchy relationship) is the cornerstone of the hierarchical annotation process, directly determining the search scope of the subgraph domain in the subtype annotation stage, and thus cannot be removed. Furthermore, we conducted a supplementary experiment. Additionally, due to the HAS_ACTIVITY_IN relationship strictly depends on PARTICIPATES_IN to connect marker genes to cell types via functional pathways, removing PARTICIPATES_IN actually ablates the entire pathway relations, so we don’t need to remove the HAS_ACTIVITY_IN separately. We integrated knowledge from the CellMarker [42] and PanglaoDB [43] databases, widely considered the “gold standard” for cell markers, into the knowledge graph as an IS_MARKER_FOR relationship. However, the inclusion of this relationship had a negative impact on scHilda’s performance. To illustrate this, we provide an example where the IS_MARKER_FOR relationship leads to an annotation failure. In Cluster 31 of the HCA-Heart dataset (True label: T cell), the KG retrieved four IS_MARKER_FOR links for ‘natural killer cell’ and only two for ‘T cell’. Seduced by the absolute quantity of these direct links, the LLM erroneously concluded: *“The presence of four distinct genes directly registered as markers for natural killer cells provides a robust phenotypic signature... The broader alignment of the marker set with the NK cell profile in the knowledge graph forms a more comprehensive explanation for the cluster’s identity.”* This phenomenon fundamentally occurs because such direct marker relationships have extremely short vector distances in the latent space learned by LLMs, prompting the self-attention mechanism to assign them overwhelmingly high probability weights and bypass the critical evaluation of complex functional pathways. The full reasoning trace for this example is provided in **Section 3 in** S1 Text**, Reasoning Traces with the Relation IS_MARKER_FOR**. When these “gold standard” data were themselves ﬂawed or incomplete, they misled the LLM’s judgment, leading to a decrease in overall performance. This result reinforces scHilda’s core design philosophy: external knowledge should serve as an aid to reasoning, not as an absolute authority, to avoid capping the model’s performance at the quality level of the external database.

### Impact of explainability output on model performance

We previously proposed in the methods section that removing the reasoning and evidence modules from scHilda’s ﬁnal output is a strategy to effectively reduce computational costs without affecting annotation accuracy. Although some studies (like CellReasoner [44]) suggest that Chain-of-Thought (CoT) [45] as an intermediate step can improve model annotation performance, scHilda’s explainability module is not a standard CoT [45], and as a presentation of the ﬁnal result, does not participate in the intermediate reasoning process. To verify our hypothesis, we conducted a comparative experiment to evaluate model performance after removing the explainability output. As shown in Fig 2D, the impact on the ﬁnal annotation accuracy was negligible. This result conﬁrms that users can ﬂexibly choose whether to generate detailed explainability reports based on their needs. In application scenarios where process transparency is not a high priority, disabling this feature can signiﬁcantly reduce API call costs with almost no sacriﬁce in annotation performance.

### scHilda shows strong robustness in annotating mixed-cell samples

**Fig 3:**
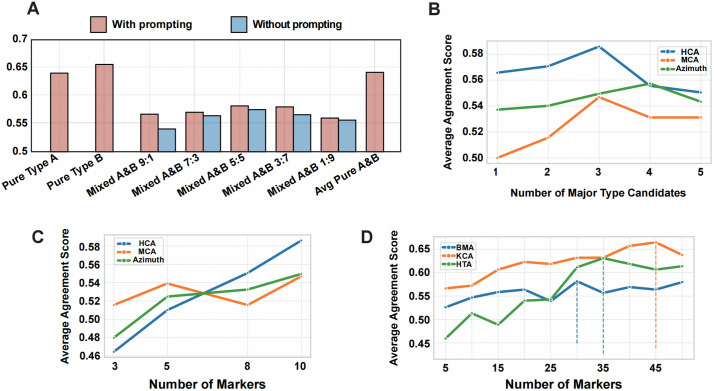
scHilda Robustness and Hyperparameter Sensitivity Analysis. (A) Performance on mixed-cell sample annotation tasks and the differences between with the prompting and without, demonstrating the strong robustness brought by the hierarchical strategy. (B) The impact of inputting different numbers of major cell type candidates (1–5) on annotation performance, showing that 3 candidates provide the best balance between accuracy and cost. (C) The impact of inputting different numbers of marker genes (3–10) on annotation performance, showing that the presence of the knowledge graph enhances the model’s robustness when fewer marker genes are available. (D) The impact of inputting different large numbers of marker genes (5–50) on annotation performance, showing that within a certain range, larger numbers of marker genes lead to better performance.

Annotating mixed-cell samples is a signiﬁcant challenge in single-cell analysis because their marker lists contain mixed signals from multiple cell types, which can easily lead to misidentiﬁcation. To evaluate scHilda’s performance on this task, we added a speciﬁc instruction to the prompt during the experiment: “Please be aware that the provided markers for each index may represent a combination of two distinct cell types.” The experimental results (Fig 3A) show that the scHilda framework exhibits strong robustness, rooted in its unique hierarchical annotation strategy. First, upon receiving input containing mixed markers for T cells (e.g., CD3D) and B cells (e.g., MS4A1), scHilda’s ﬁrst stage does not hastily make a single judgment. Instead, it proposes multiple high-probability candidate major types (e.g., “T cell lineage” and “B cell lineage”), which avoids pre- maturely compressing information into a single hypothesis. Subsequently, the framework enters the evidence integration stage, using the knowledge graph to provide objective support for all candidates. During this process, after evaluating the KG evidence, the LLM will ﬁnd that both T cell and B cell lineages have strong supporting evidence, thus conﬁrming that it is a mixed population rather than forcing an incorrect “multiple-choice” decision. Most critically, after conﬁrming the mixed components, the framework independently and separately ﬁnds the most precise subtype for each major type. For example, it will ﬁrst focus on analyzing markers related to T cells to determine their subtype, treating the B cell markers as background noise, and vice versa. This design effectively isolates interfering signals, allowing for higher precision in the subtype annotation of each component. Notably, our systematic evaluations across multiple mixing proportions reveal that even under an extreme 1:9 ratio, scHilda still maintains a robust identification accuracy by leveraging the explicit connections between the KG-provided evidence and the minor signals in the input data, thereby comprehensively demonstrating its exceptional performance in handling complex samples.

Furthermore, we investigated scHilda’s performance without explicitly prompting the model about potential mixtures. We observed a slight decrease in the detection success rate, as the LLM inherently tends to force a single consensus identity when attempting to resolve conflicting markers without prior guidance. Nevertheless, scHilda still successfully detected mixed components in most cases. This resilience is fundamentally attributed to our hierarchical strategy: the objective KG evidence effectively highlights the co-existence of distinct cell lineages, enabling the framework to maintain robust identification even without explicit prompt hints.

### Impact of the Number of Major Type Candidates

In the ﬁrst stage of the scHilda workﬂow, the LLM generates 3 most probable major cell type candidates for each cell cluster. To determine the optimal number of candidates, we tested the effect of generating 1–5 candidates on the ﬁnal annotation performance. As shown in Fig 3B, the overall performance of the model was best when the number of candidates was set to 3. Too few candidates (1 or 2) might miss the correct cell lineage due to the LLM’s inherent knowledge bias or hallucinations, while too many candidates (4 or 5) would increase the LLM’s decision-making burden. It is worth mentioning that because the scHilda framework provides evidence collected from the knowledge base for all candidates to make a judgment, in some cases, when the number of candidates was greater than 3, the results were indeed better than with 3 candidates. However, an increase in the number of candidates also led to a sharp increase in API token consumption and computational costs. Therefore, after a comprehensive trade-off of accuracy, computational overhead, and stability, we set the number of candidates to 3 as the optimal choice.

To further understand the LLM’s internal ranking distribution, we tracked the position of the true major cell type among the top 3 candidates proposed in Stage 1. In the initial prompt, the LLM is instructed to order the candidates from most likely to least likely. Across 330 evaluated cells from the Azimuth, HCL, and HCA datasets, the true label appeared as the first candidate (Top-1) in 86.97% of cases, the second in 10.91%, and the third in 2.12%, which is shown in Fig 2E.

This distribution shows that while the LLM’s primary intuition is highly accurate, the true label resides in lower-ranked positions in over 13% of instances. Relying solely on the LLM’s top-1 output would lead to a bottleneck in accuracy. This phenomenon robustly validates the necessity of scHilda’s hierarchical design—specifically the Stage 2 arbitration mechanism, which utilizes objective Knowledge Graph evidence to critically evaluate all candidates, effectively correcting the ranking biases and internal hallucinations inherent in the LLM’s initial zero-shot proposal.

### Impact of the number of input markers

We evaluated the impact of the number of input marker genes on scHilda’s annotation performance, testing scenarios with 3, 5, 8, and 10 markers (defaulting to 10). As shown in Fig 3C, more markers can provide the LLM with richer and more deﬁnitive evidence, leading to better annotation results.

To further investigate the impact of the number of input markers, we performed an extensive supplementary experiment utilizing several datasets that offer a larger number of markers [46–48] (due to the permutation-invariant nature of the self-attention mechanism inherent in LLMs nowadays, the input order of these marker genes does not affect the final annotation results unless explicitly instructed otherwise by the prompt). The results (as shown in the Fig 3D) indicate that scHilda can effectively leverage richer marker information, with its performance improving as the number of markers increases within a certain range. This is attributable to the synergistic enhancement effect of the knowledge graph. This finding contrasts with the conclusion from Hou and Ji [15], who proposed that “the top 10 marker genes yield the best performance.” We theorize that when relying solely on an LLM, an excessive number of markers may be perceived as “noise.” The scHilda framework, however, provides biological context for the markers through its knowledge graph, effectively distinguishing signal from noise and thereby converting more information into valid evidence for annotation.

We must emphasize that determining a universal “optimal” number of markers applicable to all datasets is exceedingly difficult, owing to the significant variation in gene expression profiles across different tissues and cell types. Our experiments show that the model’s performance peaks when the number of marker genes is between 35 and 45. While performance continues to fluctuate with a further increase in markers, it may also introduce excessive redundant information and interfere with the model’s judgment.

## Discussion

Cell type annotation is a key bottleneck in single-cell analysis. Current automated methods primarily rely on supervised learning or predefined marker genes, but each has its limitations. As an emerging strategy, LLMs have shown potential, but their inherent “hallucination” tendencies and the uncoordinated interaction with external knowledge limit their reliability in research applications. We conceptualize this uncoordinated interaction as the “Impedance Mismatch” problem. Specifically, although external knowledge bases provide LLMs with richer specialized information to supplement their potentially lacking internal knowledge, when the database itself is not completely accurate or complete, it may mislead the model or conflict with the LLM’s inherent cognition, thereby affecting the final judgment. In particular, using external data to score the LLM’s output is more likely to introduce the inherent biases of the information source. Therefore, scHilda shifts the role of the external knowledge base to assisting the LLM in reasoning, providing it with multifaceted and multi-angle specialized evidence. Through the hierarchical annotation strategy, scHilda effectively regularizes the model’s reasoning path and dynamically retrieves context-relevant evidence from a comprehensive knowledge graph, thereby significantly improving annotation accuracy. Experiments have shown that scHilda achieves state-of-the-art performance on multiple benchmark datasets and exhibits exceptional robustness when handling complex samples.

## Limitations

Despite scHilda’s signiﬁcant success, there is still room for performance improvement. Currently, the pathway activity and gene co-expression relationships in our knowledge graph are derived solely from HCL, a single human cell dataset. According to our interpretability analysis, most of the currently misannotated cell clusters are due to the sparse knowledge and insufﬁcient evidence in the KG, forcing the LLM to rely solely on its internal knowledge for judgment, with the outcome entirely dependent on the LLM’s upper limit. However, even based on a limited knowledge source, scHilda has demonstrated excellent results, especially showing strong annotation capabilities on datasets like MCA, which is based on mouse cells. This fully validates the superiority and excellent generalization ability of the scHilda framework.

Moreover, for practical applications, it is crucial to analyze the consistency between the generated explanations and the final predicted labels. When explanation generation is enabled, we occasionally observed explanation-answer mismatches, which typically fall into two categories stemming from the inherent generative mechanisms of current LLMs.

### Correct prediction with inaccurate explanation

Often driven by shortcut learning and post-hoc rationalization. For example, in Cluster 126 (Platelet) of the Azimuth dataset, the model correctly predicted “Platelet” but erroneously cited proliferation markers (like MKI67) as support, ignoring the biological fact that platelets are anucleate fragments incapable of mitosis. Similarly, for an Azimuth Monocyte cluster, the model hallucinated genes (e.g., MERTK, APOE) not present in the input list to justify its correct prediction. This occurs because the LLM recognizes high-frequency marker-label bindings from its training data, locks onto a high-probability answer, and then forces a rationalization to justify it, sometimes fabricating evidence.

### Correct explanation with inaccurate prediction

Typically caused by Chain-of-Thought (CoT) breakdown and conservative output strategies. For instance, in Cluster 8 (Natural Killer cell) of the Azimuth dataset, the LLM correctly reasoned that the markers were classic NK signatures and explicitly noted the absence of T-cell evidence (e.g., TCR/CD3). Yet, it finalized the label as the broader “cytotoxic T cell”. As the generated text lengthens, the model’s attention weights on earlier correct logic decay. Furthermore, Reinforcement Learning from Human Feedback (RLHF) often makes LLMs overly conservative, prompting them to output a “safe,” broader parent class when facing uncertainty at the final output stage.

While these are ubiquitous challenges in current generative AI, scHilda mitigates these issues to a large extent. By explicitly grounding the LLM’s reasoning in traceable, structured evidence from the Knowledge Graph and employing a hierarchical arbitration strategy, scHilda significantly constrains the model’s free-text hallucination space. As the fundamental reasoning stability of future baseline LLMs continues to evolve, we anticipate the consistency artifacts will naturally diminish.

## Future research

Therefore, we believe that with the expansion of the knowledge base, scHilda will exhibit even more powerful performance. Our future work will focus on further expanding and enriching scHilda’s knowledge graph. First, by integrating more diverse and cross-species public datasets (such as Tabula Sapiens [49], GTEx [34], etc.), we will significantly enhance the coverage and information density of the knowledge base. Second, we plan to extend the framework’s capabilities beyond transcriptomics by incorporating multi-omics data, such as single-cell ATAC-seq, into the knowledge graph. This will provide the large language model with deeper epigenetic regulatory context for cell annotation. Third, with the rapid advancement of spatial transcriptomics, integrating spatial neighborhood relationships and tissue microenvironment data into our graph structure will be a crucial next step to decode complex cellular interactions. Finally, exploring automated, dynamic updating mechanisms for the knowledge graph via text-mining the latest biomedical literature will ensure that the model remains at the cutting edge of biological discoveries. A continuously enhanced knowledge graph will provide stronger reasoning support for scHilda, thereby pushing its performance to new heights and providing a solid foundation for building the next generation of trustworthy and interpretable biological artificial intelligence systems.

## Supporting information

S1 Text Supplementary Texts and Tables. This file contains all supporting data and detailed analysis as follows: Section 1. Differences in reasoning traces between o3 and DeepSeek V3.2: Detailed step-by-step reasoning logs for three case studies illustrating distinct error patterns (over-extrapolation, hallucinated ID, and premature conclusion). Section 2. Reasoning Traces of Prompt Engineering Failure Cases: Examples of model reasoning logs under biased prompt strategies, demonstrating the necessity of neutral wording. Section 3. Reasoning Traces with the Relation IS_MARKER_FOR: A specific reasoning trace illustrating the potential negative impact of direct marker-to-cell-type associations in the knowledge graph. Table A. Accuracy on Benchmark Datasets: Comparison of cell type annotation accuracy between scHilda and baseline methods across multiple datasets. Table B. F1 Score on Benchmark Datasets: Comparison of F1 scores for scHilda and baseline methods across multiple datasets. Table C. Annotation performance with original or randomized marker input: Evaluation of model robustness against the input order of marker genes.
(DOCX)
