# Supplementary material for: scHilda: Hierarchical Integration of LLM with KG database for single cell type annotation
Source: PLoS Comput Biol. 2026 May 11;22(5):e1014291. doi: 10.1371/journal.pcbi.1014291 (PMC13175463; doi:10.1371/journal.pcbi.1014291)
Supplement: S1 Text — This file contains all supporting data and detailed analysis as follows: Section 1. Differences in reasoning traces between o3 and DeepSeek V3.2: Detailed step-by-step reasoning logs for three case studies illustrating distinct error patterns (over-extrapolation, hallucinated ID, and premature conclusion). Section 2. Reasoning Traces of Prompt Engineering Failure Cases: Examples of model reasoning logs under biased prompt strategies, demonstrating the necessity of neutral wording. Section 3. Reasoning Traces with the Relation IS_MARKER_FOR: A specific reasoning trace illustrating the potential negative impact of direct marker-to-cell-type associations in the knowledge graph. Table A. Accuracy on Benchmark Datasets: Comparison of cell type annotation accuracy between scHilda and baseline methods across multiple datasets. Table B. F1 Score on Benchmark Datasets: Comparison of F1 scores for scHilda and baseline methods across multiple datasets. Table C. Annotation performance with original or randomized marker input: Evaluation of model robustness against the input order of marker genes. (DOCX) [file pcbi.1014291.s001.docx]

**scHilda: Hierarchical Integration of LLM with KG Database for Single Cell Type Annotation**

Yilang Li^1, &^, Yidi Sun^2, &^, Aoyun Geng^2, &^, Junlin Xu^3^, Yajie Meng^4^, Feifei Cui^2^, Leyi Wei^5^, Quan Zou^6^, Xiulai Li^2, *^, Zilong Zhang^2, *^

*^1^School of Cyberspace Security (School of Cryptology), Hainan University, Haikou, China*

*^2^School of Computer Science and Technology, Hainan University, Haikou, China*

*^3^School of Computer Science and Technology, Wuhan University of Science and Technology, Wuhan, Hubei, China*

*^4^School of Computer Science and Artificial Intelligence, Wuhan Textile University, Wuhan, Hubei, China*

*^5^Centre for Artificial Intelligence driven Drug Discovery, Faculty of Applied Science, Macao Polytechnic University, Macao Special Administrative Region, China*

*^6^Institute of Fundamental and Frontier Sciences, University of Electronic Science and Technology of China, Chengdu, China*

*** [lixiulai01@hainanu.edu.cn](mailto:lixiulai01@hainanu.edu.cn), [zhangzilong@hainanu.edu.cn](mailto:zhangzilong@hainanu.edu.cn)

**1. Differences in reasoning traces between o3 and DeepSeek V3.2**

Detailed step-by-step reasoning logs for three case studies illustrating distinct error patterns (over-extrapolation, hallucinated ID, and premature conclusion).

**Case 1**: Over-extrapolation (Azimuth - Adipose, Cluster 4)

**Ground Truth**: monocyte (CL:0000576)

**Prediction of o3**: monocyte (CL:0000576)

**Prediction of DeepSeek V3.2**: Classical Monocyte (CL:0002057)

**Candidates**: monocyte, macrophage, neutrophil

**Input Marker**: VCAN, FCN1, SLC11A1, CSF3R, IL1B, KYNU, SAMSN1, CTSS, RNF144B, COTL1

**Reasoning Trace of o3**:

Step 1: Assess Evidence Quality. All three candidates (monocyte, macrophage, neutrophil) show equal KG support: each is a well-defined ontology term, each links the supplied markers to one functional pathway, and none shows an internal co-expression network. Therefore, the KG does not preferentially elevate any single candidate on purely structural grounds.

Step 2: Analyze Conflicts. The initial LLM analysis favored monocyte based on hallmark genes (VCAN, FCN1, SLC11A1, IL1B, CSF3R). The KG is neutral, offering no contradictory curated links for any candidate. However, in biological context VCAN and FCN1 are highly specific for classical/inflammatory monocytes, whereas macrophage-specific signatures (MERTK, APOE, C1QA/B, TREM2) and neutrophil-specific signatures (S100A8/A9, MPO, CXCR2, FCGR3B) are absent.

Step 3: Weigh Evidence and Conclude. Given that the KG does not resolve the tie, the discriminating factor becomes marker specificity: VCAN and FCN1 are directly curated for inflammatory monocytes, and SLC11A1 plus IL1B are strongly enriched in circulating monocytes. The lack of core macrophage or neutrophil markers further tips the balance toward monocyte.

Step 4: Final Verdict. Integrating the neutral KG evidence with the high specificity of the observed marker set, the most plausible major cell type is monocyte (CL:0000576).

**Reasoning Trace of DeepSeek V3.2**:

Step 1: Assess Evidence Quality. I have critically evaluated the Knowledge Graph (KG) summaries for the provided candidates (monocyte, macrophage, neutrophil). All three are well-defined ontology terms and each links the supplied markers to one functional pathway. The KG structural evidence is balanced across these broad categories.

Step 2: Analyze Conflicts. The initial hypotheses cover general myeloid lineages. However, examining the specific qualitative evidence from the marker list reveals VCAN, FCN1, SLC11A1, and IL1B. While these fall under the monocyte umbrella, VCAN and FCN1 are not just generic monocyte markers; they are canonical, highly definitive markers for the classical (inflammatory) monocyte subset.

Step 3: Weigh Evidence and Conclude. As the final arbiter, my goal is to provide the most definitive and precise biological judgment. Although the KG supports the broad 'monocyte' category, assigning a generic label ignores the high-resolution signal provided by VCAN and FCN1. The absence of non-classical markers (like FCGR3A) further solidifies this specific identity.

Step 4: Final Verdict. Integrating the KG's validation of the monocyte lineage with the exquisite specificity of the marker profile, the most accurate and definitive cell type assignment is Classical Monocyte (CL:0002057).

**Case 2**: Hallucinated Ontology ID (Azimuth - Bone Marrow, Cluster 24)

**Ground Truth**: plasma cell (CL:0000786)

**Prediction of o3**: Plasma cell (CL:0000786)

**Prediction of DeepSeek V3.2**: Plasma Cell (CL:0000980)

**Candidates**: plasma cell, memory B cell, B cell

**Input Marker**: SDC1, IGLC6, IGLV6-57, TNFRSF17, IGLV31, TNFRSF13B, IGLC7, JSRP1, FCRL5, IGKV1-5

**Reasoning Trace of o3**:

Step 1: Assess Evidence Quality. All three candidates have equal quantitative KG metrics (0 functional pathways, 0 co-expression links), but plasma cell, unlike the others, is linked in the KG to the highly distinctive markers SDC1 (CD138) and TNFRSF17 (BCMA) both manually curated, lineage-defining features for antibody-secreting cells.

Step 2: Analyze Conflicts. The initial LLM already preferred the plasma-cell hypothesis. The apparent KG neutrality (0 pathways) is not a negative signal; it simply means curated pathway data are sparse for all three. Qualitatively, however, the KG lists SDC1 and BCMA as signature markers that are essentially absent from memory- and naïve-B cell annotations. Memory-B evidence rests mainly on FCRL5, which is less specific and shared with plasmablasts, whereas the presence of SDC1 is plasma-cell-specific.

Step 3: Weigh Evidence and Conclude. The coexistence of SDC1, TNFRSF17, TNFRSF13B and a flood of immunoglobulin light-chain transcripts matches the canonical KG description of terminally differentiated plasma cells. Memory-B and general B-cell labels cannot parsimoniously explain the strong SDC1/BCMA signal.

Step 4: Final Verdict. Considering the specificity of SDC1 and BCMA to the plasma-cell lineage and the lack of similarly definitive markers for the other candidates, the most plausible major cell type for Cluster 24 is Plasma cell.

**Reasoning Trace of DeepSeek V3.2**:

Step 1: Assess Evidence Quality. Reviewing the KG summaries, all three candidates (plasma cell, memory B cell, B cell) lack robust pathway or co-expression networks (0 links). However, the KG specifically annotates SDC1 (CD138) and TNFRSF17 (BCMA) as highly distinctive, curated markers for plasma cells.

Step 2: Analyze Conflicts. The presence of SDC1 and TNFRSF17 creates a strong conflict with the memory B cell and generic B cell hypotheses. Memory B cells do not typically express SDC1 at the levels indicated by a definitive marker profile. The KG uniquely aligns these hallmark genes with the terminal antibody-secreting state.

Step 3: Weigh Evidence and Conclude. The combination of SDC1, TNFRSF17, and abundant immunoglobulin transcripts provides an overwhelming, definitive qualitative signature for plasma cells. The generic candidates cannot account for this highly specialized functional profile.

Step 4: Final Verdict. Based on the conclusive marker-to-cell-type linkages supported by the Knowledge Graph, the major cell type is definitively identified as Plasma Cell (CL:0000980)

**Case 3**: Premature Conclusion & Skipped Logic (Azimuth - Bone Marrow, Cluster 23)

**Ground Truth**: granulocyte monocyte progenitor (CL:0000557)

**Prediction of o3**: Granulocyte progenitor (CL:0000557)

**Prediction of DeepSeek V3.2**: Neutrophil (CL:0000775)

**Candidates**: Neutrophil, Eosinophil, Granulocyte progenitor

**Input Marker**: SERPINB10, RNASE3, MS4A3, PRTN3, ELANE, AZU1, CTSG, RNASE2, RETN, NPW

**Reasoning Trace of o3**:

Step 1: Assess Evidence Quality. All three candidates (Neutrophil, Eosinophil, Granulocyte progenitor) have identical, minimal KG metadata each is a valid ontology term but none show curated pathway or co-expression support. Hence, I must rely primarily on marker biology.

Step 2: Evaluate Marker Specificity. PRTN3, ELANE, AZU1 and CTSG are archetypal primary-granule enzymes expressed strongly at the promyelocyte stage of ALL granulocyte lineages. RNASE2 and RNASE3 are classic eosinophil granule ribonucleases. MS4A3 is a hallmark of granulocyte–monocyte progenitors that is lost during terminal neutrophil or eosinophil maturation.

Step 3: Resolve Conflicts. Mature neutrophils rarely co-express RNASE2/3, and mature eosinophils down-regulate ELANE/PRTN3/CTSG. Only an immature granulocytic stage before full lineage commitment is expected to co-express BOTH sets of genes together with MS4A3.

Step 4: Weigh Evidence and Conclude. The mixed primary-granule signature plus MS4A3 and the absence of terminal markers such as FCGR3B (neutrophil) or SIGLEC8/CCR3 (eosinophil) point most specifically to an early granulocyte progenitor (promyelocyte/myeloblast). This explanation reconciles all observed markers more convincingly than either mature neutrophil or eosinophil identities.", "Step 5: Final Verdict. The major cell type for cluster 23 is therefore Granulocyte progenitor (CL:0000557).

**Reasoning Trace of DeepSeek V3.2**:

Step 1: Assess Evidence Quality. The KG provides minimal metadata for the three candidates (Neutrophil, Eosinophil, Granulocyte progenitor), with 0 curated pathways or co-expression links. My definitive judgment must therefore rest on a critical evaluation of marker biology.

Step 2: Evaluate Marker Specificity. The marker list is prominently driven by PRTN3 and ELANE. As a computational biologist, I recognize these as archetypal, highly penetrant primary-granule enzymes that are defining hallmarks of the neutrophil lineage. Their strong signal heavily anchors the cluster's identity.

Step 3: Resolve Conflicts. While there are multiple granulocytic candidates proposed, the definitive presence of ELANE and PRTN3 resolves ambiguity. Eosinophils rely on distinct granule proteins (e.g., MBP, EPX), and a generic progenitor label underrepresents the clear commitment to the neutrophil program evidenced by these primary functional enzymes.

Step 4: Weigh Evidence and Conclude. The robust primary-granule signature completely supersedes the less specific alternative hypotheses. The qualitative evidence points unambiguously to a mature, committed granulocyte state.", "Step 5: Final Verdict. Relying on the definitive hallmark genes present in the profile, the most likely major cell type is Neutrophil (CL:0000775).

**2. Reasoning Traces of Prompt Engineering Failure Cases**

Examples of model reasoning logs under biased prompt strategies, demonstrating the necessity of neutral wording.

**Case 1**: LLM-Bias Failure (Azimuth - Bone Marrow, Cluster 30)

**Ground Truth**: Proliferating Natural Killer (CL:0000623)

**Input Marker**: GZMA, GZMB, TRDC, PRF1, TYMS, FGFBP2, KLRF1, CD247, GZMH, CCL4

**Prediction of** **Neutral Prompt**: Natural Killer

**Prediction of LLM-Biased Prompt**: Gamma-delta T cell

**Reasoning Trace of LLM-Biased Prompt**:

Step 1: Assess Evidence Quality. All three candidates are recognized ontology terms, but only the NK cell shows a single curated pathway in the KG. However, that pathway is generic (cytotoxicity) and does not address lineage-defining markers. The KG lists no co-expression evidence for any candidate, so coverage is sparse. Therefore, I must also consider the intrinsic specificity of the submitted markers.

Step 2: Analyze Conflicts. The key conflict is that the KG gives marginally more quantitative support to ‘natural killer cell’ (one pathway), while the initial hypothesis highlights TRDC, which is essentially absent from NK biology and is lineage-defining for γδ T cells. The KG’s lack of γδ-specific pathway data is likely a coverage gap rather than negative evidence, because TRDC is not expected to appear in NK pathways at all.

Step 3: Weigh Evidence and Conclude. TRDC, together with the pan-T-cell CD247 and the full cytotoxic granzyme/perforin module, is a highly specific signature for γδ T cells. NK cells lack T-cell-receptor genes, and αβ CD8 T cells would show TRAC/TRBC rather than TRDC. Thus, even though the KG provides a solitary generic pathway for NK cells, the lineage-specific marker TRDC outweighs that weak, non-specific support.

Step 4: Final Verdict. Considering marker specificity and recognizing the KG’s limited coverage for γδ T-cell pathways, the most plausible major cell type for this cluster is ‘gamma-delta T cell’ (CL:0000798)

**Case 2**: **KG-Bias Failure** (Azimuth - Bone Marrow, Cluster 19)

**Ground Truth**: Progenitor B / pro-B cell (CL:0000826)

**Input Marker**: CYGB, UMODL1, EBF1, MME, VPREB1, DNTT, IGLL1, UHRF1, BLNK, AGPS

**Prediction of** **Neutral Prompt**: Progenitor B cell

**Prediction of KG-Biased Prompt**: B cell (generic)

**Reasoning Trace of KG-Biased Prompt**:

Step 1: Assess Evidence Quality. All three candidates are valid ontology terms, but only the generic ‘B cell’ shows a curated functional-pathway link (1 pathway) connecting the submitted marker set to this lineage. The other two candidates have zero pathway or co-expression support in the KG.

Step 2: Analyze Conflicts. The initial LLM preferred the more granular ‘pre-B cell’ because the marker panel (EBF1, VPREB1, IGLL1, DNTT, BLNK, MME) indeed typifies an early B development stage. However, the KG contains no explicit functional or network evidence for ‘pre-B cell’ or ‘common lymphoid progenitor’, whereas it does for the broader ‘B cell’. This suggests the KG’s curated knowledge is stronger for the major lineage rather than the developmental sub-stage.

Step 3: Weigh Evidence and Conclude. Because the task is to decide on the most likely major cell type, and the KG provides its only positive functional pathway support to ‘B cell’, the balance of curated evidence favors the broader category. The absence of pathway/network links for the more specific terms weakens those hypotheses in this context.

Step 4: Final Verdict. Therefore, integrating the KG’s stronger support and the requirement to output a major cell type, the cluster is best annotated as ‘B cell’ (CL:0000236).

**3. Reasoning Traces with the Relation IS_MARKER_FOR**

A specific reasoning trace illustrating the potential negative impact of direct marker-to-cell-type associations in the knowledge graph.

**Cluster**: HCA - Human Heart, Cluster 31

**Ground Truth**: T cell (CL:0000084)

**Input Marker**: SKAP1, ARHGAP15, B2M, FAM65B, CCND3, PTPRC, ANKRD44, PARP8, THEMIS, BCL2

**Prediction**: natural killer cell

**Reasoning Trace**:

Step 1: Assess Evidence Quality. The KG summaries indicate valid ontology terms for both natural killer cell and T cell. For the natural killer cell, the KG retrieves IS_MARKER_FOR relationships for four genes in the list (PTPRC, B2M, ARHGAP15, BCL2). For the T cell, the KG identifies IS_MARKER_FOR links for two genes (THEMIS, SKAP1), as well as PARTICIPATES_IN edges connecting these two genes to T-cell receptor signaling pathways.

Step 2: Analyze Conflicts. The initial LLM analysis leaned toward the T cell hypothesis. However, the retrieved KG evidence shows a higher volume of direct marker matches for the natural killer cell lineage. While THEMIS and SKAP1 are linked to T-cell signaling pathways, natural killer cells and T cells are closely related lymphoid lineages that share significant signaling machinery and activation states.

Step 3: Weigh Evidence and Conclude. The presence of four distinct genes directly registered as markers for natural killer cells provides a robust phenotypic signature. The pathway involvement of THEMIS and SKAP1 can be parsimoniously explained by the shared developmental biology of cytotoxic lymphocytes. The broader alignment of the marker set with the NK cell profile in the knowledge graph forms a more comprehensive explanation for the cluster's identity.

Step 4: Final Verdict. Integrating the predominant marker associations retrieved from the knowledge graph, the most plausible major cell type is natural killer cell (CL:0000623).

**Regular metric (Accuracy and F1 score) on methods**

4. Table A in S1 Text. Accuracy on Benchmark Datasets: Comparison of cell type annotation accuracy between scHilda and baseline methods across multiple datasets.

Table A in S1 Text: Accuracy on Benchmark Datasets

| Datasets | scHilda | CellTypeAgent | GPTCellType |
| --- | --- | --- | --- |
| Azimuth | 0.4023 | 0.3831 | 0.2508 |
| BCL | 0.5556 | 0.5000 | 0.2500 |
| HCA | 0.4468 | 0.4787 | 0.3535 |
| HCL | 0.4000 | 0.2459 | 0.3455 |
| coloncancer | 0.3333 | 0.2857 | 0.1667 |
| lungcancer | 0.6000 | 0.6000 | 0.0000 |

5. Table B in S1 Text. F1 Score on Benchmark Datasets: Comparison of F1 scores for scHilda and baseline methods across multiple datasets.

Table B in S1 Text: F1 Score on Benchmark Datasets

| Datasets | scHilda | CellTypeAgent | GPTCellType |
| --- | --- | --- | --- |
| Azimuth | 0.1042 | 0.1038 | 0.0713 |
| BCL | 0.4167 | 0.0714 | 0.1538 |
| HCA | 0.2001 | 0.2001 | 0.1547 |
| HCL | 0.2095 | 0.0392 | 0.1398 |
| coloncancer | 0.1667 | 0.0000 | 0.0909 |
| lungcancer | 0.4286 | 0.0000 | 0.1765 |

**Annotation performance with original or randomized marker input**

6. Table C in S1 Text. Annotation performance with original or randomized marker input: Evaluation of model robustness against the input order of marker genes.

Table C in S1 Text: Annotation performance with original or randomized marker input

| Datasets | Original | Randomized |
| --- | --- | --- |
| KCA | 0.6378 | 0.6359 |
| BMA | 0.5798 | 0.5855 |
| HTA | 0.6137 | 0.6162 |
